# Quantitative Bottom-Up Glycomic Analysis of Polysaccharides in Food Matrices Using Liquid Chromatography–Tandem Mass Spectrometry

**DOI:** 10.1021/acs.analchem.2c03707

**Published:** 2022-12-21

**Authors:** Nikita P. Bacalzo, Garret Couture, Ye Chen, Juan J. Castillo, Katherine M. Phillips, Naomi K. Fukagawa, Carlito B. Lebrilla

**Affiliations:** † Department of Chemistry, University of California—Davis, Davis, California 95616, United States; ‡ Virginia Tech, Blacksburg, Virginia 24061, United States; § Beltsville Human Nutrition Research Center, USDA Agricultural Research Service, Beltsville, Maryland 20705, United States; ∥ Department of Biochemistry and Molecular Medicine, University of California—Davis, Davis, California 95616, United States

## Abstract

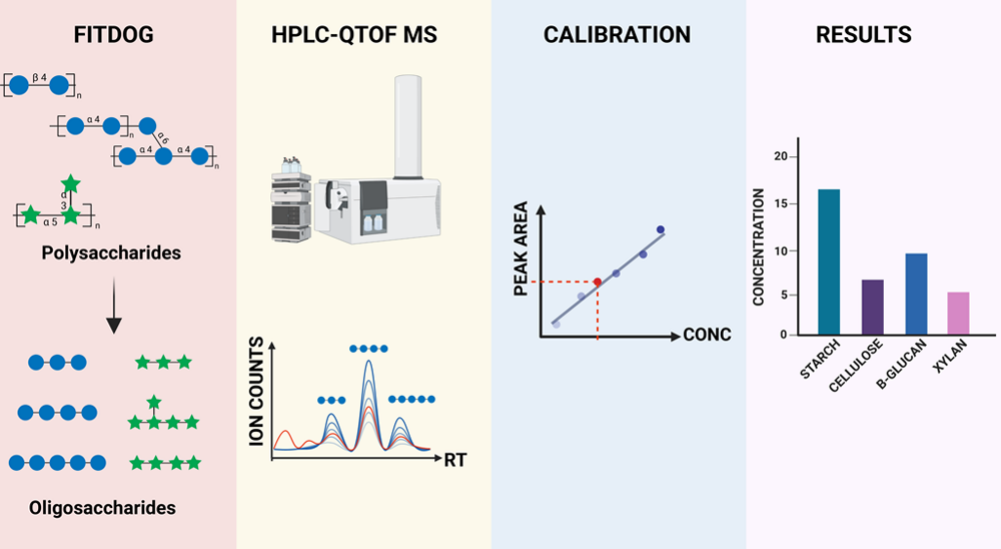

Carbohydrates are the most abundant biomolecules in nature, and specifically, polysaccharides are present in almost all plants and fungi. Due to their compositional diversity, polysaccharide analysis remains challenging. Compared to other biomolecules, high-throughput analysis for carbohydrates has yet to be developed. To address this gap in analytical science, we have developed a multiplexed, high-throughput, and quantitative approach for polysaccharide analysis in foods. Specifically, polysaccharides were depolymerized using a nonenzymatic chemical digestion process followed by oligosaccharide fingerprinting using high performance liquid chromatography–quadrupole time-of-flight mass spectrometry (HPLC-QTOF-MS). Both label-free relative quantitation and absolute quantitation were done based on the abundances of oligosaccharides produced. Method validation included evaluating recovery for a range of polysaccharide standards and a breakfast cereal standard reference material. Nine polysaccharides (starch, cellulose, β-glucan, mannan, galactan, arabinan, xylan, xyloglucan, chitin) were successfully quantitated with sufficient accuracy (5–25% bias) and high reproducibility (2–15% CV). Additionally, the method was used to identify and quantitate polysaccharides from a diverse sample set of food samples. Absolute concentrations of nine polysaccharides from apples and onions were obtained using an external calibration curve, where varietal differences were observed in some of the samples. The methodology developed in this study will provide complementary polysaccharide-level information to deepen our understanding of the interactions of dietary polysaccharides, gut microbial community, and human health.

## Introduction

Carbohydrates are the most abundant class of biomolecules in nature;^1^ however, their analysis remains challenging. Polysaccharides in particular remain difficult to analyze because of their structural and compositional diversity. Food carbohydrates play an important role in human health, both directly (e.g., absorbed free sugars and products of gastrointestinal hydrolysis of starch) and indirectly from the impact of nondigestible components (“dietary fiber”) on nutrient absorption and on the gut microbiome.^2^ More recently, the effect of undigested polysaccharides (and oligosaccharides) in shaping and modulating the community of microbes in the human gut and the effect on human health have been recognized and are the subject of widespread research efforts.^3,4^ While endogenous human carbohydrate-active enzymes (CAZymes) are limited in function, gut microbes have a vast array of CAZymes that can potentially degrade polysaccharides and ferment them into secondary metabolites.^5^ Different polysaccharide compositions and structures affect the gut microbiota in various ways owing to the taxonomical and functional diversity of these microbes.^6^ Overall, changes in the gut microbiome induced by exposure to various polysaccharides can in turn induce metabolic and physiological changes in their host.^7,8^ Detailed characterization of the food carbohydrates, specifically their chemical structures, is indispensable in establishing the relationship between food and health but analytical methods for comprehensive polysaccharide characterization are lacking.^9^

Starch and nonstarch polysaccharides in foods are typically measured indirectly by enzymatic-gravimetric methods (e.g., AOAC 991.43, AOAC 2011.25, AOAC 2017.16) to obtain food composition data. When specific polysaccharides are characterized, they are typically extracted from biological sources first, and then fractionated by different buffers based on solubility. These fractions are then subjected separately to monosaccharide and linkage analyses and the polysaccharide structures are inferred.^10,11^ NMR techniques can be performed to confirm the primary structures of the purified polysaccharides.^12,13^ Although this approach can provide an in-depth structural analysis, it is impractical for large-scale analysis of many foods and food products. NMR has also been recently used for absolute quantitation of some common polysaccharides. However, this specific method required the molar stoichiometry of monosaccharides in the mixture.^14^ Other methodology has involved the use of CAZymes to deduce polysaccharide structure, where oligosaccharide products from selective enzymatic digestion are in turn characterized using chromatography and/or mass spectrometry (MS).^10,15^ However, each enzyme reaction often requires optimization, rendering the method highly laborious with very low throughput. Monoclonal antibodies have also been developed and used to detect specific polysaccharides in plant tissues. This assay is typically performed in a microarray format where the extracted polysaccharide fractions are immobilized on multiple substrates to allow antibody binding.^16,17^ While the method can have high throughput, limitations include the cost and availability of the antibodies, and extensive matrix effects of native samples.

**Figure 1:**
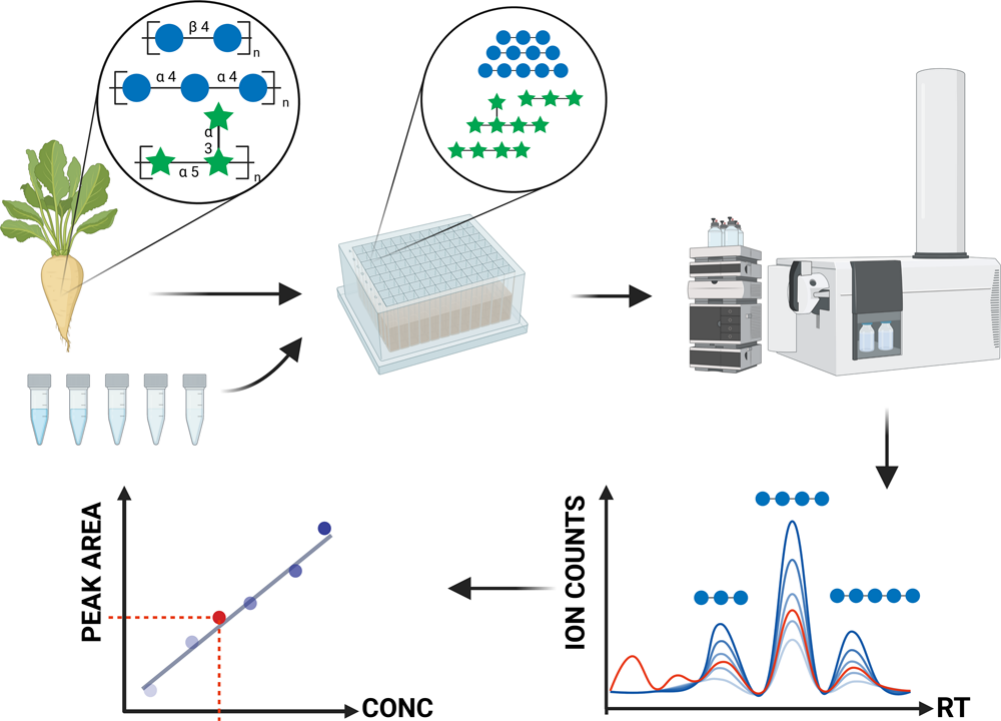
Overview of the analytical method for the identification and quantitation of polysaccharides using FITDOG and HPLC-QTOF profiling of the resulting oligosaccharides. The peak areas and the use of external calibration curves provided absolute quantitation.

To address the lack of a widely applicable and high-throughput method for quantitative polysaccharide analysis in foods, we have developed a method using a bottom-up glycomics approach (Figure 1). Polysaccharide identification was based on the generation of characteristic oligosaccharides that were produced using Fenton chemistry in a reaction called “Fenton’s Initiation Towards Defined Oligosaccharide Groups” (FITDOG).^18,19^ The oligosaccharides were used as fingerprinting features to identity and quantitate polysaccharides based on chromatographic and tandem MS (MS/MS) analysis, where MS/MS provides compositional analysis of the oligosaccharides and chromatographic retention times facilitate further identification, with peak areas used for quantitation of the parent polysaccharides. The methodology presented here significantly improves on our previously published workflow. The ability to simultaneously measure absolute concentration of nine polysaccharides in a single method is unprecedented. This approach was validated using standards and was applied to a variety of food types to identify and quantitate polysaccharides, in terms of both relative and absolute concentrations.

## Methods

### Materials and Reagents

The following polysaccharide standards were used to generate the fingerprinting library and to construct the calibration curve for absolute quantitation (purchased from Megazyme (Bray, Ireland) unless otherwise stated): chitin (shrimp shells, BioReagent grade, Sigma-Aldrich), starch (corn, analytical grade, Sigma-Aldrich), cellulose (microcrystalline powder, extra pure, average particle size 90 μm, ACROS Organics), arabinan (sugar beet pulp, purity > 95%), mannan (ivory nut seeds, purity > 98%), galactan (potato fiber, purity > 85%), xylan (beechwood, purity > 95%), xyloglucan (tamarind seeds, purity > 95%), and β-glucan (barley flour, purity ∼ 95%). Various fruits, vegetables, and herbs were prepared for method testing and were purchased from local grocery stores in Davis, CA, USA. Apples and onions were procured and analyzed for the USDA Food Data Central Foundation Foods database (https://fdc.nal.usda.gov) from different retail stores in the Beltsville, MD and Blackburg, VA areas in 2020. Apples were analyzed with skin but without the stem and core, and onions were analyzed without skin. Preparation of homogenates in liquid nitrogen and storage of the prepared subsamples was as described previously.^20^

### Food Sample Preparation

Food samples were processed in the following steps: lyophilization, pulverization into powder, precipitation with 80% ethanol, drying, resuspension in water, and bead homogenization before being plated into the 96-well reaction plate. Multiplexed quantitation of polysaccharides was enabled by pooling several polysaccharide standards together. Calibration standards were prepared by weighing and pooling the polysaccharide powders into vials, suspending the pooled standards with water, homogenizing and heating with beads, and then finally plating into the reaction plate.

### Depolymerization Reaction Using Fenton’s Reagent

We have previously optimized this reaction to yield reproducible and diverse oligosaccharides.^19^ The reaction mixture consisted of 95% (v/v) 44 mM sodium acetate buffer (adjusted to pH 5.20 with glacial acetic acid), 5% (v/v) of 30% (v/v) H_2_O_2_, and 73 μM Fe_2_(SO_4_)_3_·5H_2_O. To each of the well, an aliquot of 100 μL of sample or standard mixture was transferred, then 900 μL of the reaction mixture was added and allowed to react for 1 h at 100 °C using an incubator oven without shaking. The reaction was quenched by adding 500 μL of freshly prepared 2 M NaOH, followed by glacial acetic acid (61 μL) for neutralization. The resulting oligosaccharides were then reduced by incubation with an equal volume of 1.0 M NaBH_4_ for 1 h at 65 °C, followed by isolation and cleanup using sequential solid-phase extractions (SPE) in a 96-well plate format. Samples were cleaned up first with C18 SPE and then with porous graphitized carbon (PGC) SPE. The recovered and cleaned-up oligosaccharides were completely dried by centrifugal vacuum evaporator and stored at −20 °C until analysis.

### Liquid Chromatography–Mass Spectrometry

Samples were reconstituted in 100 μL of Nanopure water prior to analytical separation, which was carried out using an Agilent 1260 Infinity II HPLC (Agilent Technologies, Santa Clara, CA, USA). Chromatographic separation was performed on a 150 mm × 1 mm Hypercarb column from Thermo Scientific (5 μm particle size). The column compartment was set at 40 °C. A binary gradient was employed and consisted of solvent A: (3% (v/v) ACN, 0.1% FA in water) and solvent B: (90% ACN, 0.1% FA in water). A 45 min gradient with a flow rate of 0.132 mL/min was used: 3–25% B, 0–15 min; 25–25% B, 15–18 min; 25–99% B, 18–30 min; 99–99%B, 30–32 min; 99–3% B, 32–34 min; 3–3% B, 34–45 min.

HPLC was coupled to Agilent 6530 Accurate-Mass Q-TOF mass spectrometer (Agilent Technologies, Santa Clara, CA, USA). The MS detector was run in the positive mode with the following electrospray source parameters: drying gas temperature = 150 °C. drying gas flow rate = 11 L/min, fragmentor = 175 V, skimmer = 60 V, octupole 1 RF = 750 V. Acquisition mode was set to data-dependent mode, where top 5 most abundant precursor ions were selected for fragmentation. Dynamic exclusion was enabled for 30 s. The acquisition rate was set to 0.63 spectra/s. For tandem MS fragmentation, a linear function for collision energy (CE), where CE = 1.45*(*m*/*z*)-3.5, was employed.

### Data Analysis

For annotation of oligosaccharide peaks from food samples, an in-house script was used (see example in Supplementary Figure S1 and Table S1). Raw data was first converted to MGF (Mascot Generic Format) files to be parsed by GlycoNote, a Python script previously developed in our laboratory for automated glycan composition annotation from tandem MS spectra (https://github.com/MingqiLiu/GlycoNote). Chromatographic peak area abundances (based on extracted precursor ion chromatograms) were obtained using MassHunter Quantitative Analysis for Q-TOF (version 10.1, Agilent Technologies), where peaks were manually integrated. Peak area table was exported to Microsoft Excel for quantitation. For each polysaccharide, peak areas of the top 3 most abundant oligosaccharides were averaged and used for the calibration curve. At least five points were used in the linear regression fit (equal weighing) and the intercepts were forced to zero.

## Results and Discussion

By using a nonenzymatic bottom-up approach for polysaccharide analysis, we have developed a high-throughput, multiplexed, and quantitative method to analyze polysaccharide in food samples. Multiplexing was enabled by the FITDOG reaction in which multiple polysaccharides with diverse chemical structures were depolymerized into distinct oligosaccharides products. Polysaccharides standards were reacted using FITDOG and the oligosaccharides were used to construct a fingerprint library.^18,19^ By using external calibration curves, we have further extended the application to absolute quantitation in the more complex food samples, chosen to contain different types and amounts of polysaccharides. Quantitation of polysaccharides using the proposed methodology (Figure 1) was validated for recovery using the commercially available polysaccharide standards.

### Generation of Fingerprint Profile for the Polysaccharides

**Figure 2:**
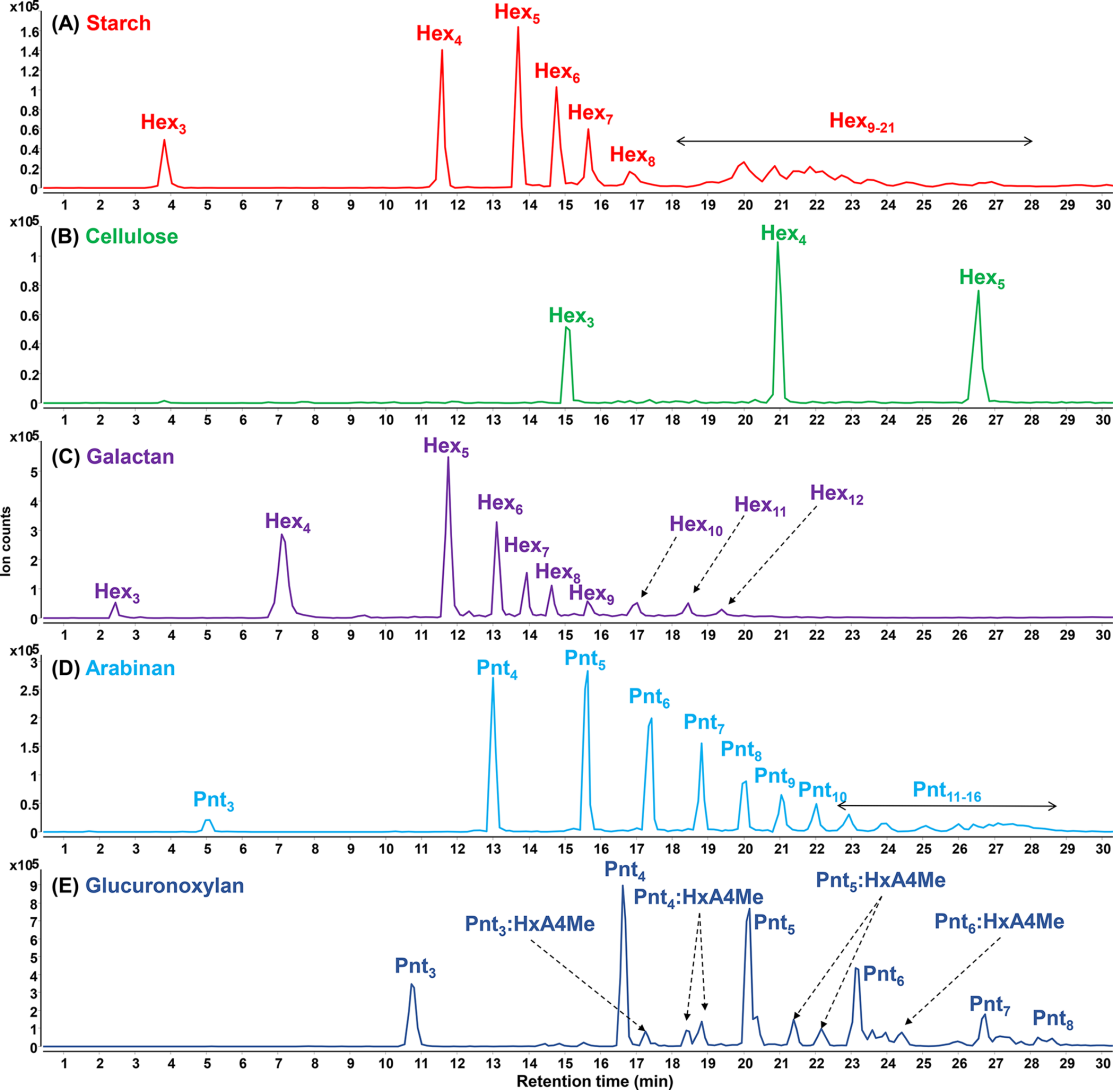
Example chromatograms showing oligosaccharide products from FITDOG reactions of each polysaccharide. Hex = hexose, Pnt = pentose, HxA = hexuronic acid, 4Me = 4-*O*-methyl.

Example oligosaccharide chromatograms from FITDOG-reacted polysaccharide standards are shown in Figure 2. Starch is composed of amylose and amylopectin, where amylose is a linear homopolymer of glucose connected with an α(1 → 4) linkage, while amylopectin is similar to amylose with branching points with an α(1 → 6) linkage.^1^ The FITDOG reaction with starch yielded oligosaccharides of varying degrees of polymerization (DP), ranging from 3 up to 21. Both amylose and amylopectin standards gave similar oligosaccharide profiles after reaction with FITDOG. Cellulose is another linear homopolymer of glucose connected with a β(1 → 4) linkage. The difference in anomeric configuration between starch and cellulose oligosaccharides resulted in distinct oligosaccharide profiles. Galactan polysaccharide is composed of β(1 → 4)-linked galactose residues and is usually attached as a side branch in pectin polysaccharides.^21^ Galactan oligosaccharides resulting from the FITDOG reaction ranged from DP 3 up to DP 12. Arabinan is another domain present in pectin polysaccharides, where the backbone is comprised of α(1 → 5)-arabinofuranose residues.^22^ Xylan is a plant polysaccharide with a linear backbone of β(1 → 4)-xylose and occasionally with branches of glucuronic acid residues.^23,24^ The glucuronic acid residues are further typically *O-*methylated at the C4 position. Xylan oligosaccharides, including the methylated glucuronic acid residues, were detected using the FITDOG workflow. Oligosaccharide chromatogram profiles of other polysaccharides (mannan, chitin, β-glucan, xyloglucan) are shown in Supplementary Figure S2. The complete oligosaccharide fingerprint library is summarized in Supplementary Table S2.

### Validation of Quantitation Using Oligosaccharide and Polysaccharide Standards

Quantitative results were validated by using commercially available standards. First, commercial oligosaccharide standards were pooled and serially diluted at different concentrations and injected in the HPLC-QTOF to determine the instrument response with respect to concentrations (Supplementary Figure S3). This demonstrated that the HPLC-QTOF method generated proportional changes in the peak area in response to analyte concentration and can be amenable to quantitation. Different compounds gave distinct relative responses as measured by the slopes of the fitted linear regression. This observation highlighted the need for generating a separate calibration curve for each analyte of interest.

**Figure 3:**
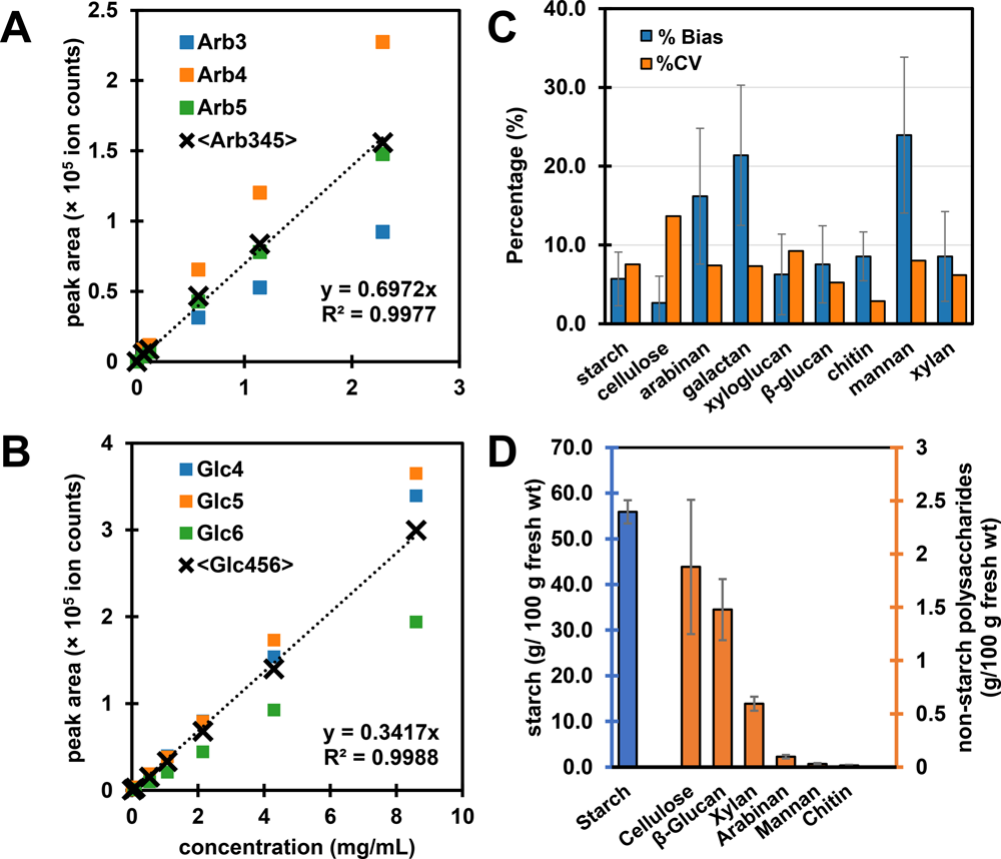
Quantitative results from FITDOG analysis of polysaccharide standards. External calibration curves for (A) linear arabinan standard and (B) starch. Arb = arabinose, Glc = glucose. (C) Accuracy (% bias) and reproducibility (% CV) based on mixtures of polysaccharide standards. %CV was computed based on three method replicates. (D) FITDOG results for polysaccharides in a standard reference material (Fortified Breakfast Cereal, NIST SRM 3233). Right vertical axis corresponds to nonstarch polysaccharides (chitin, mannan, arabinan, xylan, β-glucan, cellulose), while left vertical axis corresponds to starch values.

Quantitation of polysaccharides was evaluated using calibration curves prepared by subjecting polysaccharide standards (starch, cellulose, β-glucan, xyloglucan, mannan, galactan, arabinan, xylan, chitin) to the FITDOG analysis. Several pooled mixtures of polysaccharide standards were prepared and serially diluted to generate the calibration curve standards. To get a more representative quantitation metric, chromatographic peak areas of the top three most abundant unique oligosaccharides from each polysaccharide were averaged and was used for the calibration curves. For example, the linear arabinan standard yielded 13 oligosaccharides that could be used for quantitation. From these arabinan oligosaccharides, Arb_3_, Arb_4_, and Arb_5_ were the most abundant and their peak areas were averaged and used for the calibration curve. Representative calibration curves for the linear arabinan standard and for starch are shown in Figure 3A and B. This calibration process was done for all the other polysaccharides (Table 1, Supplementary Figure S4). Overall, most calibration curves were linear (*r*^*2*^ > 0.99), except for chitin (*r*^2^ = 0.98). Among the polysaccharides, chitin had the highest slope while cellulose had the lowest slope. The method detection limit (MDL) was estimated based on the lowest concentration of standard reacted which gave an averaged peak area signal-to-noise ratio (S/R) value >3. Chitin and arabinan had the lowest MDL (∼55 μg/mL or ∼0.22 wt %/wt dry basis). The linear ranges spanned approximately 2 orders of magnitude for all polysaccharides.

**Table 1:** Calibration Curve Parameters for the Absolute Quantitation of Polysaccharides Using the Quantitative FITDOG Method

polysaccharide	*r*^2^	slope	MDL (μg/mL)	MDL (%wt/wt, dry)	S/R @ MDL
β-glucan	0.999	55325	96	0.38	2.7
chitin	0.979	117476	55	0.22	6.1
mannan	0.995	35637	89	0.36	5.1
xylan	0.995	51848	103	0.41	10.0
arabinan	0.998	69723	57	0.23	5.3
galactan	0.996	24987	532	2.13	8.0
xyloglucan	0.997	26912	542	2.17	7.5
cellulose	0.997	9783	350	1.40	3.7
starch	0.999	34165	538	2.15	16.6

To verify the quantitative approach, several pooled mixtures of standard polysaccharides were prepared, analyzed, and quantified using the proposed calibration method. The accuracy of the method was quantified by percent (%) difference between the measured and expected concentration (based on nominal concentration of the test mixtures), while the reproducibility was demonstrated by percent coefficient of variation (CV) based on three technical replicates taken through the entire method (Figure 3C). The accuracy ranged from 5% to 25% bias, while the reproducibility ranged from 2% to 15% CV. In terms of accuracy, arabinan, galactan, and mannan values had the most deviation (>15%) from the expected concentration, while starch, β-glucan, xyloglucan, and xylan had the least deviations (<10%). Six out of nine polysaccharides quantified had <10% bias. Furthermore, the workflow was highly reproducible with %CV of less than 10% for all polysaccharides except cellulose (14% CV).

To test method performance on a food matrix, NIST SRM 3233 Fortified Breakfast Cereal (National Institute of Standards and Technology, Gaithersburg, MD, USA) was analyzed in triplicate (Figure 3D). The Certificate of Analysis (COA) for this material includes reference values for total carbohydrates by difference (79.23 ± 1.04 g/100 g), total free sugars (16.07 ± 1.53 g/100 g), and low molecular weight soluble dietary fiber (LMW SDF, 3.07 ± 0.62 g/100 g). Total carbohydrates by difference include all forms of carbohydrates, including free sugars, oligosaccharides, and all polysaccharides. Subtracting total free sugars and the LMW SDF fraction from the total carbohydrates will provide an estimate of the polysaccharide fraction of the cereal standard.^25^ Total assayed polysaccharides from FITDOG (sum of starch, cellulose, mannan, β-glucan, chitin, arabinan, xylan) was 60.02 ± 2.63 g/100 g and this was within the expected range for total polysaccharide estimated from COA values (Table 2).

**Table 2:** Summary of Results for Fortified Breakfast Cereal (NIST SRM® 3233) Based on Certificate of Analysis (COA) and the FITDOG Method

COA		FITDOG	
attribute	g/100 g fresh wt	polysaccharide	g/100 g fresh wt
total carbohydrates	79.23 ± 1.04	starch	55.92 ± 2.54
total sugars	16.07 ± 1.53	cellulose	1.88 ± 0.63
LMW SDF	3.07 ± 0.62	β-glucan	1.48 ± 0.29
		xylan	0.59 ± 0.07
		arabinan	0.10 ± 0.02
		mannan	0.03 ± 0.01
		chitin	0.01 ± 0.01
**Estimated polysaccharide**	60.08 ± 1.95	**total polysaccharide**	60.02 ± 2.63

To assess the reproducibility of the various steps of the workflow, five commercial polysaccharide standards were pooled, reacted, and injected to the instrument. Each step of the workflow was done with 6–7 replicates and at least 29 oligosaccharides were monitored from the five polysaccharides (Supplementary Figure S5). In this experiment, samples from the previous step of the method were aliquoted and pooled to serve as the replicates for the next step. The largest variations were observed with replicates taken through the entire assay starting from the FITDOG reaction step. Less variations were observed from replications in the subsequent steps of the workflow, namely in the NaBH_4_ reduction and SPE cleanup. Overall, the validation experiments using standards demonstrated the accuracy and reproducibility of the FITDOG workflow for multiplexed, high-throughput, absolute quantitation of polysaccharides.

### Quantitation of Polysaccharides in Food Samples

Representative chromatograms for select food samples are shown in Supplementary Figure S6. In both artichoke samples (Figure S6A,B), the oligosaccharide fingerprints showed cellulose and xylan, but starch was only in the inner leaves sample. The avocado seed (Figure S6C) showed high amount of starch, while the avocado skin (Figure S6D) had glucuronoxylan and cellulose. Avocado seed has been previously shown to contain high amounts of starch.^26^

**Figure 4:**
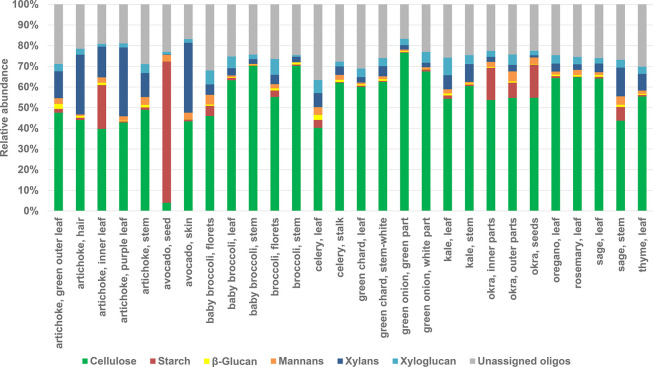
Relative quantitation of polysaccharides in food samples based on extracted ion chromatogram peak area abundances.

Relative quantitation results for single measurements of 13 different foods are shown in Figure 4. To get a diverse set of polysaccharides and to demonstrate the generality of the method, these foods were also partitioned to several anatomical parts, including some nonedible parts, for analysis. The relative abundance was based on peak areas of extracted ion chromatograms of the oligosaccharides resulting from the FITDOG reaction. Broccoli stems and green onion had the highest relative amount of cellulose. Avocado seed had the highest amount of starch out of all the samples analyzed. Okra and some artichoke parts showed appreciable amounts of starch. Xylans were also detected in lower amounts in several artichoke samples, avocado skin, and sage stem. Unassigned oligosaccharides referred to peaks identified as oligosaccharides based on tandem mass spectra but were not matched to any polysaccharide based on retention times from the oligosaccharide fingerprinting library. These unassigned oligosaccharides accounted for 20–30% relative abundance based on peak area across all samples and were mostly Hex_n_:Pnt_1_ and Hex_n_:Pnt_1_:HxA_1_ oligosaccharides.

**Figure 5:**
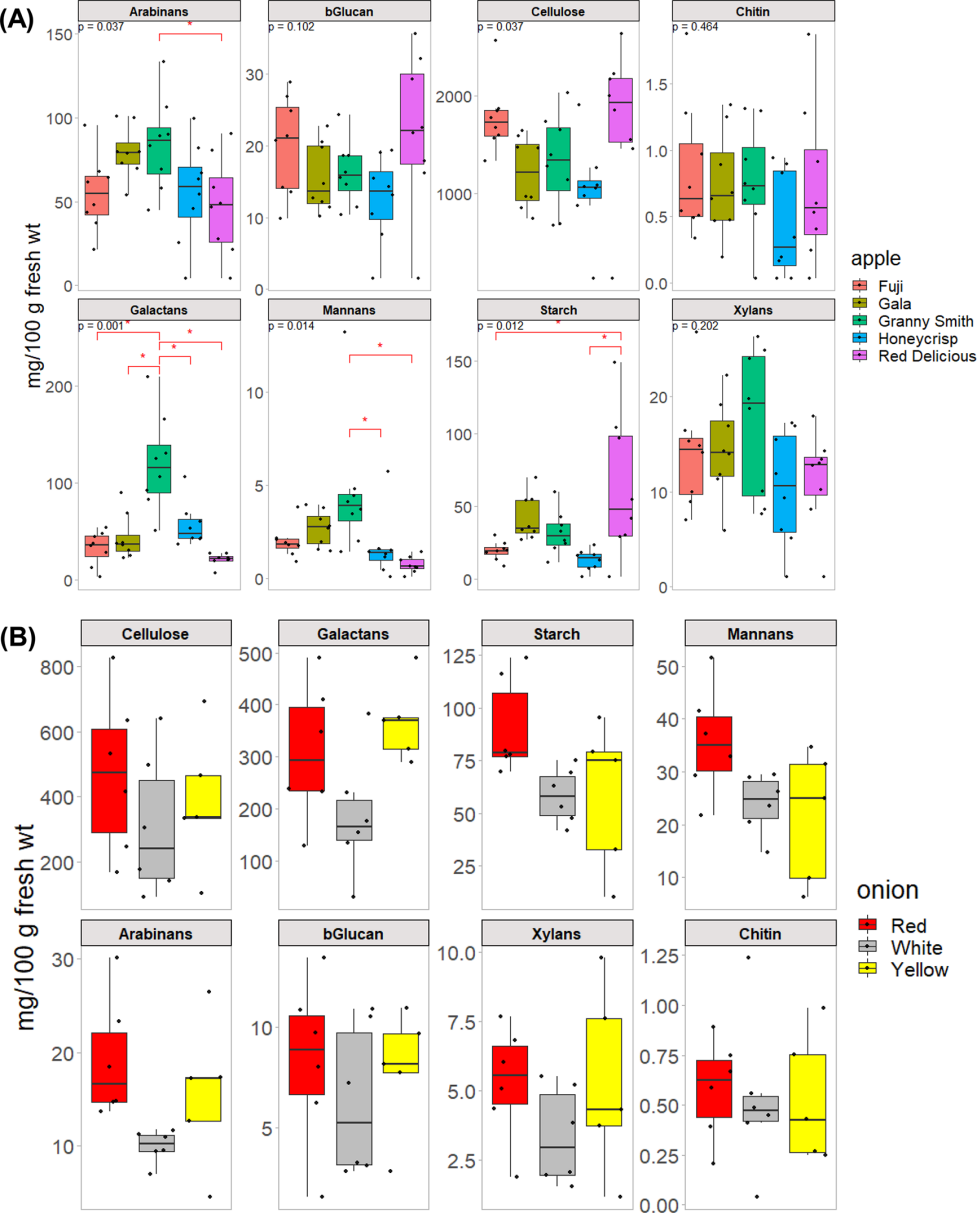
Boxplot graphs showing content of different polysaccharide types among varieties of (**A**) apples (7–8 independent retail samples of each of Fuji, Gala, Granny Smith, Honeycrisp, Red Delicious varieties), and (**B**) onions (6 independent retail samples of each of white, red, and yellow varieties). *P*-values shown in (**A**) were false-discovery-rate adjusted *p*-values from ANOVA, while the red asterisk (*) denotes post hoc Tukey’s test *p* < 0.05.

Finally, using the external calibration curve, the absolute quantitation workflow was then applied to several sample sets consisting of apples and onions (Figure 5). The apple set consisted of five varieties, where each had 7–8 samples obtained from different sources. Three varieties of onions were analyzed, each with 6 samples obtained from different sources.

Among the apples, cellulose was the most abundant polysaccharide which ranged from 1 to 2% (wt/wt by fresh weight). Galactans, arabinans, and starch had values < 0.2%. Based on analysis of variance (ANOVA) done for each analyte, the five most abundant polysaccharides (cellulose, arabinan, galactan, mannan, and starch) were statistically significant between the five varieties analyzed (FDR-adjusted *p* < 0.05). Additionally, post hoc comparison tests using Tukey’s test revealed some differences between these five varieties. For example, Granny Smith apple had significantly higher amounts of galactan, arabinan, and mannan compared to the other four varieties. Red Delicious apple had the highest amount of starch, while Fuji and Honeycrisp had the lowest starch content. Although cellulose had a significant *p*-value from the ANOVA, the post hoc comparison tests did not show any significant pairwise comparisons between the five varieties. It has been previously shown that apples have significant amounts of cellulose.^27^ Arabinans and galactans were observed at appreciable amounts in the apples analyzed, which was expected based on previously published data on the carbohydrate characterization of apples. Pectic polysaccharides in apples are known to contain both arabinan and galactan branches.^28^

Among the onions, no significant differences were found across all polysaccharides between the three varieties analyzed. White onion had consistently lower amounts of polysaccharides. Cellulose was again the most abundant polysaccharide, followed by galactan and starch. Previously published data has shown that onion cell walls are comprised mostly of pectins, hemicelluloses, and cellulose.^29,30^

The apple and onion samples demonstrated that results from our workflow corroborated with existing data on the expected polysaccharides found in these samples. However, the quality of quantitative data obtained from our workflow is unprecedented in terms of scale, coverage, and throughput. Previously published papers on food carbohydrate analysis involved complex fractionation schemes, and quantitation from these studies is often limited to monosaccharide and glycosidic linkage analyses.

### Limitations and Future Work

The reported approach has not been optimized yet to detect and quantitate some other food polysaccharides, such as fructans (e.g., inulin, levan) and other pectic polysaccharides, such as polygalacturonans and rhamnogalacturonans. Galacturonans are anionic polysaccharides containing galacturonic acid residues.^1^ These anionic oligosaccharides can potentially be analyzed better in negative mode ionization. Additionally, as discussed in the text before, the recoveries for some polysaccharides could still be improved. Standard addition method can be used, however, throughput will slightly decrease due to the number of samples necessary for standard addition. Using internal standards can be further explored, although stable-isotope-labeled polysaccharides are generally uncommon and can be prohibitively expensive. Nevertheless, we envision that this approach will lead to new techniques to be developed to analyze polysaccharides, especially due to its compatibility with being conducted in a multiplexed, high-throughput, semiautomated workflow.

## Conclusion

A high-throughput method enabling accurate and reproducible qualitative and quantitative characterization of polysaccharides in food samples was successfully demonstrated using a bottom-up glycomics approach. The method is suitable for quantitation of common food polysaccharides (e.g., starch, cellulose, mannans, arabinans, xylans, galactans, β-glucan, xyloglucan, chitin) and for comparisons among different foods and different samples of the same food.

The FITDOG reaction and the subsequent steps of the assay were optimized to be done in a 96-well plate format, increasing the throughput and making it amenable to automation. In the current setup, two plates (corresponding to as many as 168 food samples and 24 calibration standards) can be reacted and prepared in parallel within 2 days and can be run on the instrument for 6 days (45 min/sample). This throughput is an improvement from conventional food composition analysis that is normally done in single vial preparations.

The FITDOG method provides a more comprehensive characterization of the types and absolute amounts of different polysaccharides in food samples, compared to traditional standard enzymatic-gravimetric methods for quantitation of total starch and nonstarch polysaccharides, or “dietary fiber”, in foods. This specificity was demonstrated using NIST SRM 3233 (fortified breakfast cereal) where a more detailed polysaccharide composition of the sample was determined. In comparison, traditional methods only provided bulk measurements with minimal structural information. With this additional layer of information, dietary fiber composition can be further specified. In the case of apples, for example, we have shown statistically significant differences between different varieties in their arabinan, galactan, mannan, and starch contents. This kind of resolution in carbohydrate structures can provide valuable input to other research fields, such as precision breeding in agriculture and personalized diet formulations in nutrition.

Furthermore, the FITDOG method complements the other high-throughput methods we have reported,^31−34^ such as monosaccharide and glycosidic linkage analyses. This suite of glycomics-based methodologies can advance research studies on food composition, including processing effects on food carbohydrates as well as the effect of dietary carbohydrate components on the gut microbiome and their impact on health outcomes.

## Data Availability

Identification and quantitation results, together with the raw mzML files were deposited in GlycoPost (ID GPST000285).

## Supporting Information Available

Methods; example of automatically annotated tandem MS spectrum; chromatogram profiles of reacted polysaccharide standards; linear response of oligosaccharides; calibration curves for all polysaccharides quantitated; reproducibility assessment for the entire workflow; example chromatograms of food samples (PDF)
Example of isomeric separation; complete oligosaccharide fingerprinting library; example of automated tandem MS annotation output (XLSX)
