# Supplementary material for: Quantitative Bottom-Up Glycomic Analysis of Polysaccharides in Food Matrices Using Liquid Chromatography–Tandem Mass Spectrometry
Source: Anal Chem. 2022 Dec 21;95(2):1008–15. doi: 10.1021/acs.analchem.2c03707 (PMC9850401; doi:10.1021/acs.analchem.2c03707)
Supplement: Supplementary file 1 — ac2c03707_si_001.pdf [file ac2c03707_si_001.pdf]

SUPPLEMENTARY INFORMATION

Quantitative bottom-up glycomic analysis of polysaccharides in food matrices using liquid chromatography – tandem mass spectrometry

Nikita P. Bacalzo Jr. [1], Garret Couture [1], Ye Chen [1], Juan J. Castillo [1], Katherine M. Phillips [2], Naomi K. Fukagawa [3], Carlito B. Lebrilla [1,4]

Author Affiliations

[1] Department of Chemistry, University of California - Davis, Davis, CA 95616, USA

[2] Virginia Tech, Blacksburg, VA, USA

[3] Beltsville Human Nutrition Research Center, USDA Agricultural Research Service, Beltsville, MD, USA

[4] Department of Biochemistry and Molecular Medicine, University of California - Davis, Davis, CA 95616, USA

SUPPLEMENTARY MATERIALS

Supplementary Methods

Supplementary Figures

Figure S1. Example of a GlycoNote-annotated tandem mass spectrum of an Hex_8 oligosaccharide from amylose standard.

Figure S2. Chromatogram profiles of oligosaccharide products from polysaccharide standards reacted with FITDOG. (**A**) mannan, (**B**) β-glucan, (**C**) xyloglucan, and (**D**) chitin.

Figure S3. Peak areas of oligosaccharides plotted against injection concentration. Monosaccharide legend: blue circle = glucose, yellow circle = galactose, red triangle = fucose, orange star = xylose, green pentagram = arabinose.

Figure S4. Examples of calibration curves of polysaccharide using the quantitative workflow presented in the paper.

Figure S5. Reproducibility for each step of the quantitative analysis of polysaccharides.

Figure S6. Examples of annotated chromatograms (oligosaccharide profiles) of various food samples. (**A**) green outer leaves from artichoke, (**B**) inner leaves from artichoke, (**C**) avocado seed, and (**D**) avocado skin. Y-axes are in ion counts.

Supplementary Tables

Table S1. Example of isomeric separation of Hex_5 isomers using porous graphitic carbon (PGC) as analytical column.

Table S2. Oligosaccharide (reduced) fingerprinting library for identification and quantitation of polysaccharides using the FITDOG workflow. Retention time values were based on the LC conditions described in the Methods section. Oligosaccharide compositions were deduced from tandem mass spectra fragmentation pattern.

Table S3. Example of GlycoNote annotation output.

Supplementary Methods

Materials and reagents

Sodium acetate ($Na(CH_3CO_2)$), hydrogen peroxide (30% H_2O_2), sodium hydroxide (NaOH), iron(III) sulfate pentahydrate ($Fe_2(SO_4)_3{\cdot}5H_2O$), chitin (shrimp shells, BioReagent grade), and starch (corn, analytical grade) were purchased from Sigma-Aldrich (St. Louis, MO, USA). Cellulose (microcrystalline powder, extra pure, average particle size 90 µm) was purchased from ACROS Organics. Linear arabinan (sugar beet pulp, purity > 95%), mannan (ivory nut seeds, purity > 98%), galactan (potato fiber, purity > 85%), xylan (beechwood, purity > 95%), xyloglucan (tamarind seeds, purity > 95%), and β-glucan (barley flour, purity ~95%) were purchased from Megazyme (Bray, Ireland). Sodium borohydride ($NaBH_4$, powder, > 99%), trifluoroacetic acid (TFA, LC-MS grade) and formic acid (FA, LC-MS grade) were purchased from Fisher Scientific (Belgium, UK). Acetonitrile (ACN, HPLC grade) was purchased from Honeywell (Muskegon, MI). Nanopure water (18.2 MΩ-cm) was used for all experiments. Various fruits, vegetables, and herbs were prepared for method testing and were purchased from local grocery stores in Davis, CA, USA. The products were selected to represent ones containing a range of polysaccharide (and other saccharides) types and levels. Apples (Red Delicious, Honeycrisp, Granny Smith, Gala, Fuji) and onions (red, yellow, white) were procured and analyzed for the USDA Food DataCentral Foundation Foods database (https://fdc.nal.usda.gov) from different retail stores in the Beltsville, MD and Blackburg, VA areas in 2020. Eight samples of each food/variety were obtained, with each sample being approximately 1-1.5 kg total. Apples were analyzed with skin but without stem and core, and onions were analyzed without skin. Preparation of homogenates in liquid nitrogen and storage of the prepared subsamples was as described previously.[21] Solid-phase extraction cartridges (C18 and PGC) in 96-well plate format were purchased from Glygen Corporation

(Columbia, MD, USA). Reaction plates in deep 96-well format (Nunc™ 96-Well Polypropylene DeepWell™ Storage Plates) were purchased from Thermo Scientific (Waltham, MA, USA).

Food sample preparation

At least 5.0 g of fresh food sample was weighed in a 50-mL screwcap tube, frozen, and freeze-dried (BenchTop Pro, SP Scientific, Warminster, PA, USA) for at least 48 hr. Moisture content was determined from the fresh weight and the dried weight after freeze drying (residual moisture was not measured and was assumed to be zero). Dried food samples were then ground into powder using 3.2-mm stainless steel beads and homogenized using the Bead Ruptor Elite Bead Mill Homogenizer (Omni International, Kennesaw, GA, USA). Each dried and ground food sample was weighed (25 mg), suspended in 1.00 mL 80% ethanol in a 1.5-mL screwcap tube, homogenized using the bead mill homogenizer, and centrifuged at 15,000 rcf for 15 min. Supernatant was discarded and the pellet residue was further washed twice using 1.00 mL 80% ethanol using the same conditions as the first wash. Ethanol-washed pellet was dried in a centrifugal vacuum evaporator. Dried pellets were then suspended in 1.00 mL water, homogenized with 0.9-mm stainless steel beads, heated at 100 ^{o}C for 1 hr using an incubator oven (OF-01E, Jeio Tech, Daejeon, Republic of Korea) without shaking, and then homogenized again with the bead mill homogenizer. A 100-µL aliquot was plated into 96 deep-well plate for the FITDOG reaction. From this step onwards, all steps were carried out in 96-well plate format, allowing for a rapid throughput and scalable method.

Calibration standards preparation

Multiplexed quantitation of polysaccharides was enabled by pooling several polysaccharide standards together. Three mixtures of polysaccharides were prepared by the following scheme: mixture 1 contains arabinan, galactan, β-glucan; mixture 2 contains xylan, mannan, xyloglucan, chitin; mixture 3 contains starch and cellulose. Each polysaccharide was weighed (~ 10 mg) in a 7-mL polypropylene vial and each mixture was suspended in 5-mL water. Pooled mixtures were homogenized with 0.9-mm stainless steel beads in a bead mill homogenizer, heated at 100 ^{o}C for 1 hr, and then homogenized again with the bead mill homogenizer. Calibration curve standards were prepared by serial dilutions of the pooled mixtures. From these, 100-μL aliquots were transferred to 96 deep-well reaction plate, together with the processed food samples for the FITDOG reaction. Mixtures used for validation were made from the same pooled mixtures but diluted at different concentrations. To assess the reproducibility of each step of the workflow, a pool of five polysaccharides (starch, cellulose, arabinan, xylan, chitin at ~2 mg/mL of each) was prepared similarly in water, homogenized, heated at 100 ^{o}C for 1 hr, and then homogenized again with the bead mill homogenizer. This pooled mixture was plated in 9 wells to assess the overall method variability. In each subsequent steps of the procedure ($NaBH_4$ reduction, clean-up, instrument injection), several aliquots from the previous steps were pooled and were used as new replicates for the next steps.

Solid phase extraction (SPE) clean-up

Reduced oligosaccharides were cleaned up with C18 SPE first, then porous graphitized carbon (PGC) SPE. Each step of the SPE protocol was carried out with a centrifuge (Centrifuge 5810-R, Eppendorf, Hamburg, Germany) with deep-well plate rotor (1,200 rcf for 1 min). For the

C18 SPE, cartridges were washed first with ACN (200 µL, 2×), then water (200 µL, 2×). The sample solution was then loaded (400 µL, 2×) and flow-through was collected. For the PGC SPE, cartridges were primed with water (400 µL, 1×), then 80% ACN, 0.1% (v/v) TFA (400 µL, 1×) and finally water (400 µL, 1×). The eluent from C18 SPE was loaded (400 µL, 2×) and washed with water (400 µL, 6×). The oligosaccharides were then eluted with 400 µL 40% ACN with 0.05% (v/v) TFA in a 0.8-mL 96-well collection plate (Abgene™ 96 Well 0.8mL Polypropylene Deepwell Storage Plate, Thermo Scientific). The recovered eluent was completely dried by centrifugal vacuum evaporator and stored at -20 ºC until analysis.

Liquid chromatography using porous graphitic carbon (PGC)

The retention mechanism of PGC is influenced mainly by the planarity and hydrophilicity of the analytes.[22] PGC had been previously shown to be very effective in resolving isomeric carbohydrate structures and so it is the most appropriate separation column for this workflow.[23,24] The capability of PGC to resolve isomeric oligosaccharides was shown with Hex_5 oligosaccharides (**Supplementary Table S1**). The different Hex_5 oligosaccharides have varying monosaccharide and linkage compositions and they were all resolved in the chromatographic dimension. For example, cellulose oligosaccharides with β(1→4)-Glc linkage have more planar structure than amylose[1], and therefore were retained more in the PGC column, as demonstrated by higher retention time values. Among the Hex_5 oligosaccharides with β(1→4) linkage listed in **Supplementary Table S1**, mannopentaose and galactopentaose oligosaccharides were less retained than cellopentaose, most probably due to the axial hydroxyl group orientations at C2 and C4 positions in mannose and galactose, respectively.

Data analysis

The FITDOG reaction was used to generate distinct oligosaccharides from parent polysaccharides. Individual polysaccharide standards were prepared at ~2.00 mg/mL in water, homogenized using the bead mill homogenizer, and then heated at 100 ^{o}C for 1 hr. Polysaccharides were then reacted using the FITDOG process and the resulting oligosaccharides were used to construct the fingerprint library (**Supplementary Table S2**). Tandem mass fragmentation spectra were used to manually assign monosaccharide class compositions to each oligosaccharide peak. Chromatographic retention times and accurate masses were used to match sample oligosaccharides to the library.

For annotation of oligosaccharide peaks from food samples, an in-house script was used. Raw data was first converted to MGF (Mascot Generic Format) files to be parsed by GlycoNote, a Python script previously developed in our laboratory for automated glycan composition annotation from tandem MS spectra (https://github.com/MingqiLiu/GlycoNote). The script generates a combinatorial library of oligosaccharides from an input of possible monosaccharide class compositions. Tandem mass spectra from each sample were filtered based on precursor ion *m*/*z* values generated from the combinatorial library. Additional diagnostic ions could also be included to filter out non-oligosaccharide spectra. Tandem mass spectral peaks were annotated based on commonly observed fragment ions of oligosaccharides (A_i, B_i, C_i, X_i, Y_i, Z_i) resulting from collision-induced dissociation (CID) fragmentation. Lastly, identification results are false discovery rate-controlled by implementing a target-decoy strategy. The script outputs several files, including image files for each of the annotated tandem mass spectra (**Supplementary Figure S1**), and a summary table which lists all the oligosaccharides identified (**Supplementary Table S3**). GlycoNote is especially useful in large batch analysis. For this specific method, mass tolerances

of 20 and 50 ppm were used for the precursor and fragment ions, respectively. The output list of compounds was filtered to >50% coverage based on intensity and monosaccharide sequence.

For the label-free relative quantitation, oligosaccharides were first assigned to their parent polysaccharide using the fingerprint library. Peak areas from the MS1 chromatograms of these oligosaccharides were summed for each polysaccharide. Relative abundances were then derived from the normalized peak area sum for each sample. Label-free relative quantitation is usually employed in other LC-MS-based -omics methodologies because of its ease and simplicity.[25,26] MS1-based relative quantitation can be used to compare across samples, but within-sample comparisons are generally not accurate due to differences in the ionization of different compounds.

Absolute quantitation using external calibration curves were done in Microsoft Excel. For each polysaccharide, peak areas of the top 3 most abundant oligosaccharides were averaged and used for the calibration curve. The range of the calibration curve varied between different polysaccharides. The highest calibrator ranged from at least 1.00 to 8.50 mg/mL and then these were serially diluted as follows: 2×, 4×, 8×, 40×, 80×. At least five points were used in the linear regression fit (equal weighing) and the intercepts were forced to zero.

Further statistics and visualization were done with R programming language. For the absolute quantitation of apples and onions, multiple one-way analysis of variance (ANOVA) was done for each polysaccharide. *P*-values were adjusted for multiple testing using Benjamini-Hochberg (also called false-discovery rate adjustment or FDR-adjusted). Pairwise post hoc mean comparisons were done using Tukey's test.

Supplementary Figures

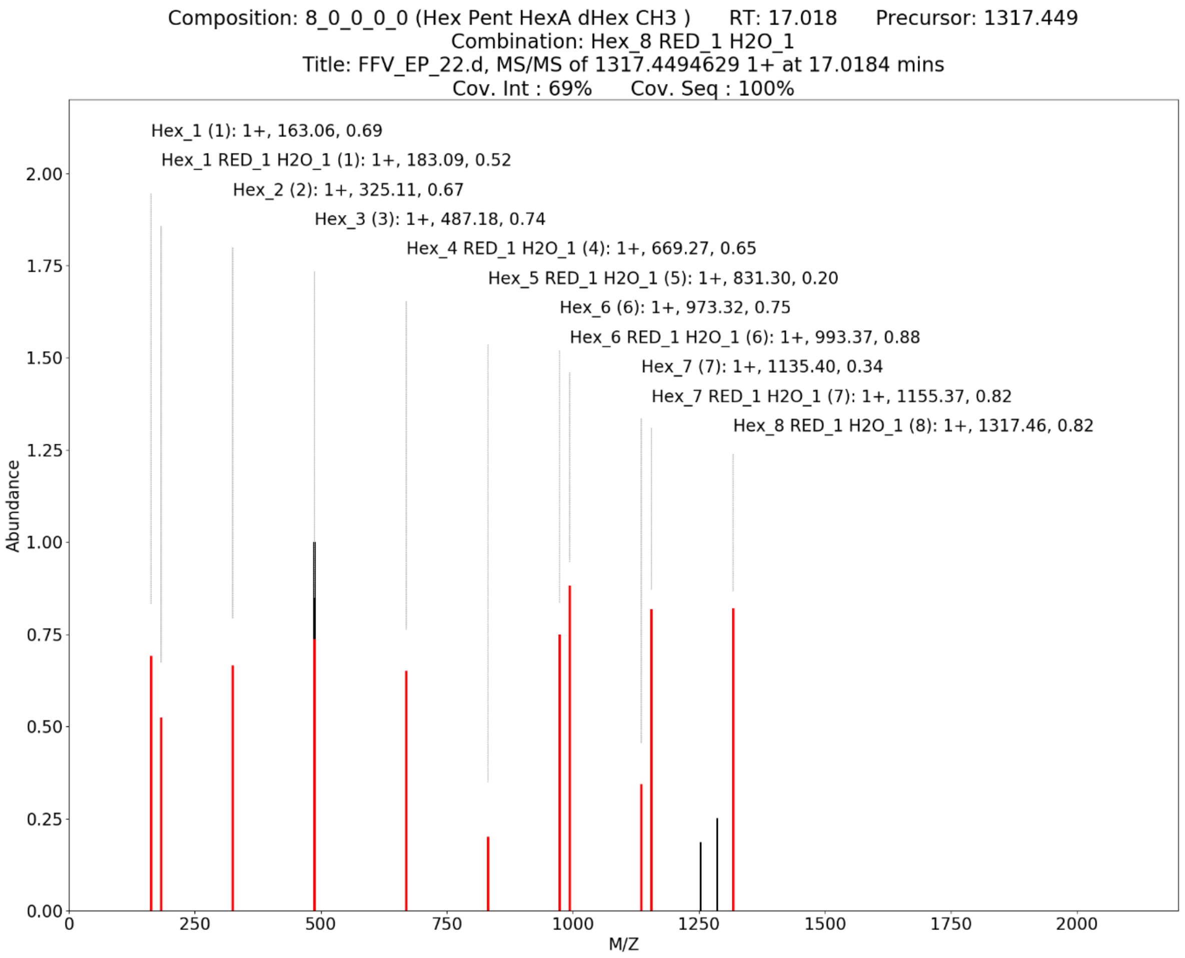

Figure S1. Example of a GlycoNote-annotated tandem mass spectrum of an Hex_8 oligosaccharide from the amylose standard.

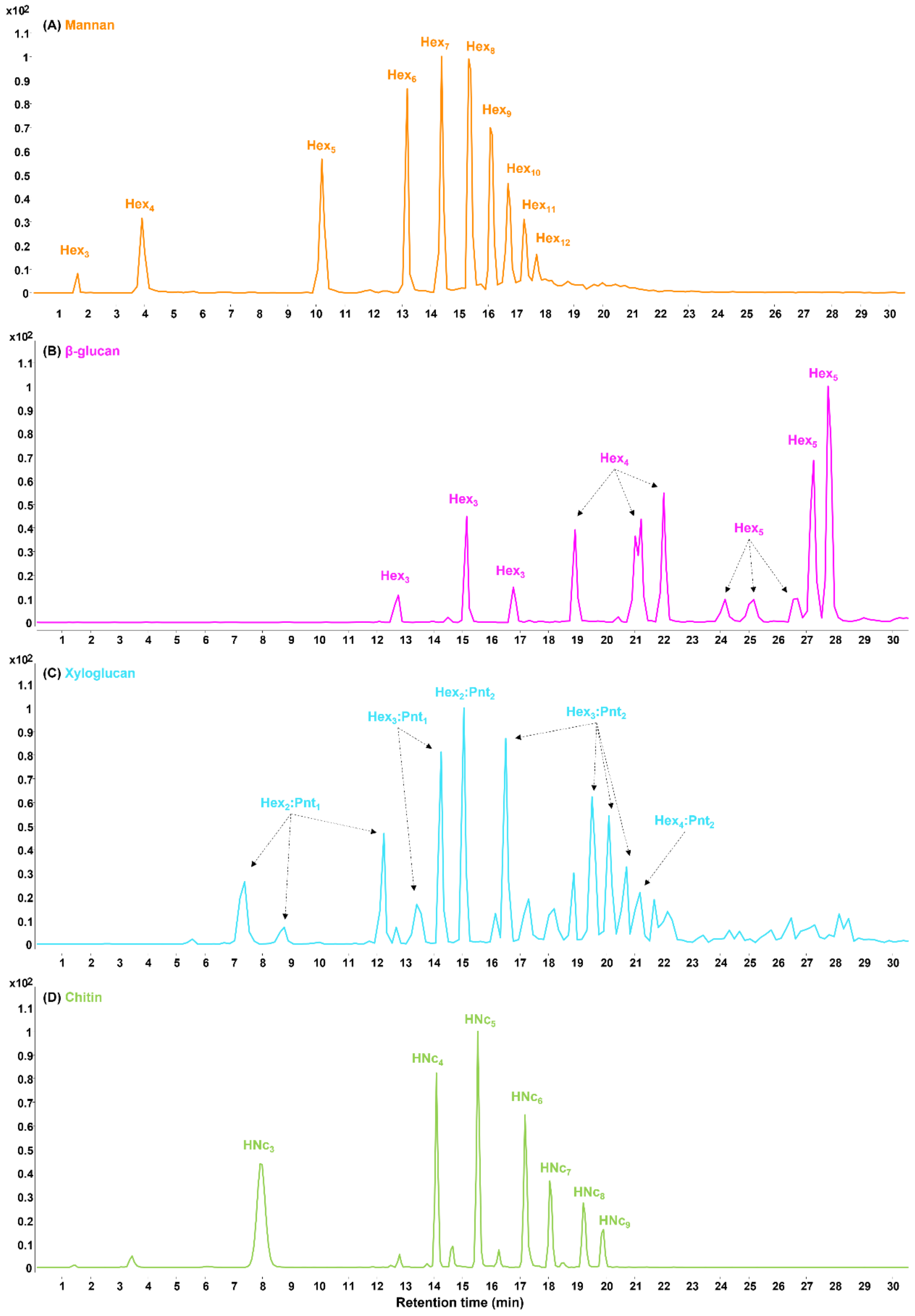

Figure S2. Chromatogram profiles of oligosaccharide products from polysaccharide standards reacted with FITDOG. (**A**) mannan, (**B**) β-glucan, (**C**) xyloglucan, and (**D**) chitin.

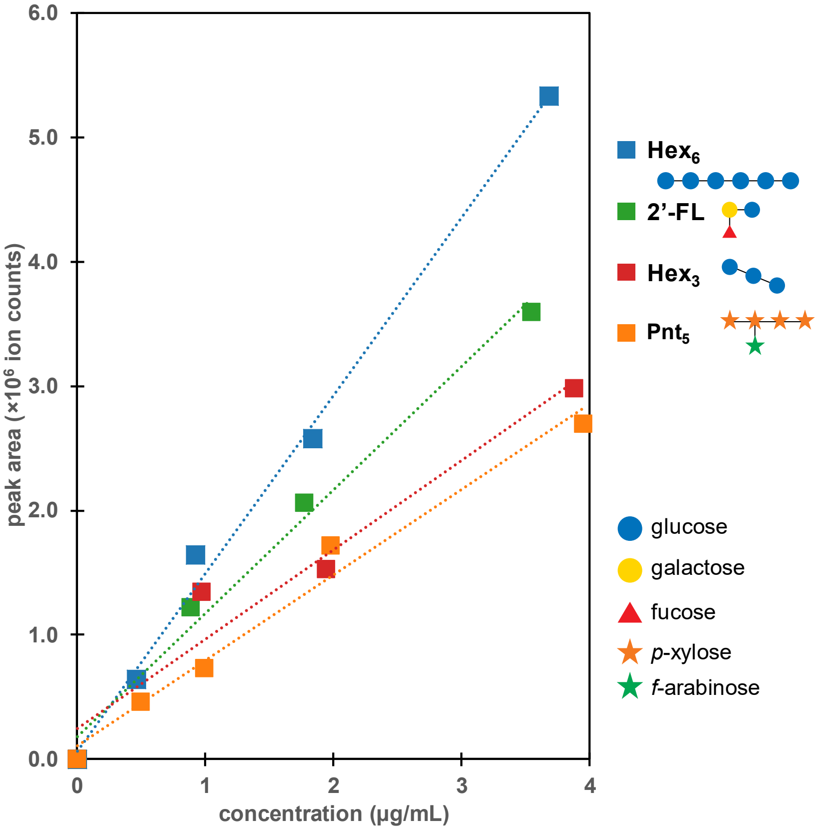

Figure S3. Peak areas of oligosaccharides (Hex_6 = maltohexaose, 2'-FL = 2'-fucosyllactose, Hex_3 = isomaltotriose, Pnt_5 = 3^3-α-L-arabinofuranosyl-xylotetraose) plotted against injection concentration. Monosaccharide legend: blue circle = glucose, yellow circle = galactose, red triangle = fucose, orange star = xylose, green pentagram = arabinose.

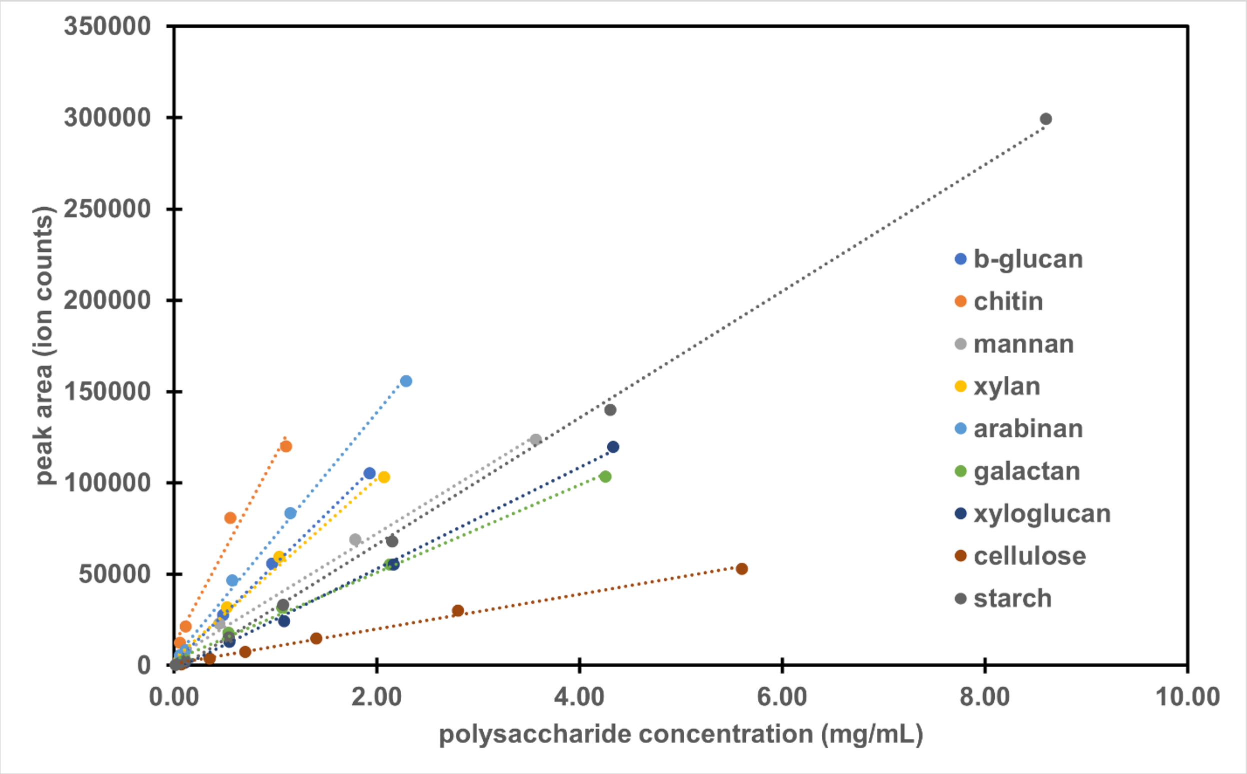

Figure S4. Examples of calibration curves of polysaccharide using the quantitative workflow presented in the paper.

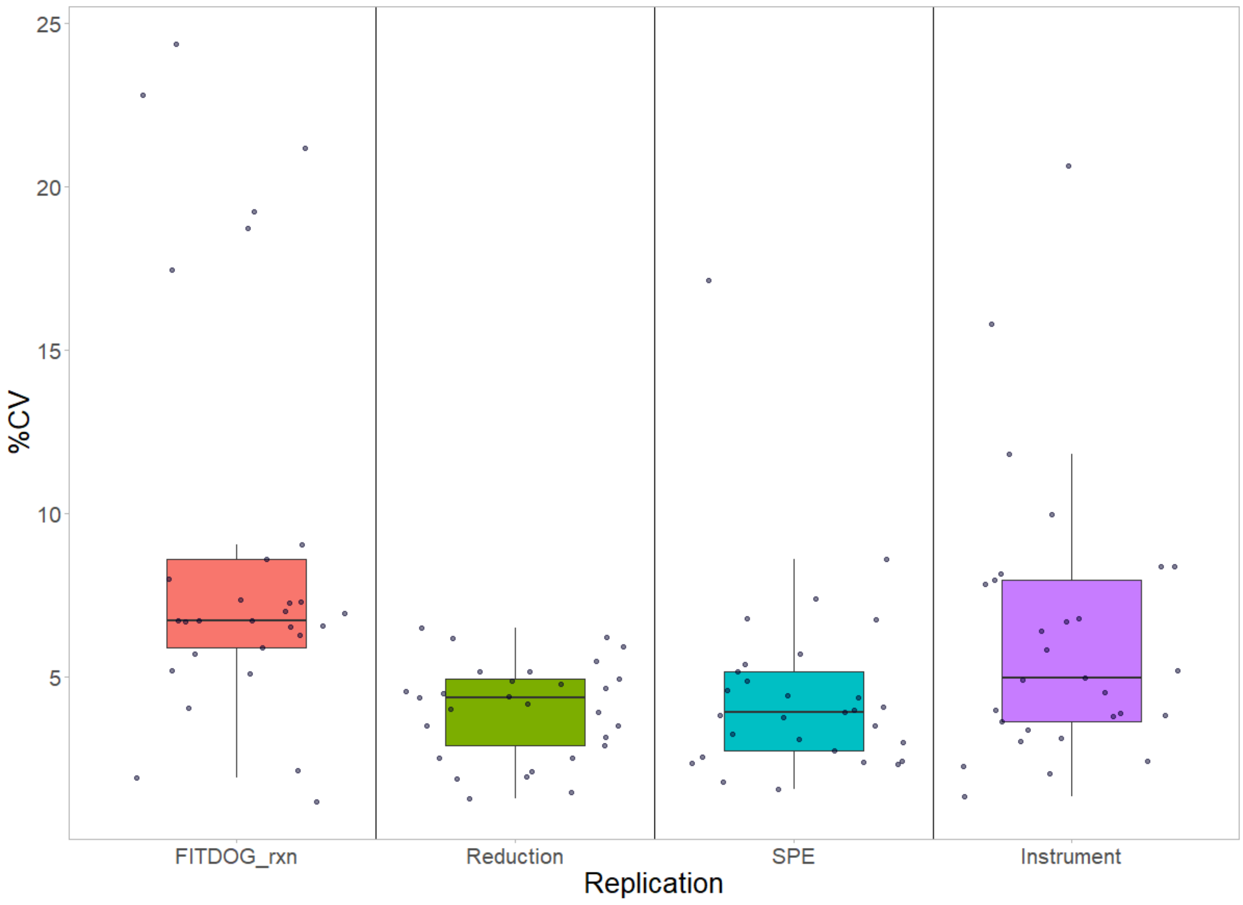

Figure S5. Reproducibility for each step of the quantitative analysis of polysaccharides. Each point corresponds to an oligosaccharide and each step had 6-7 replicates.

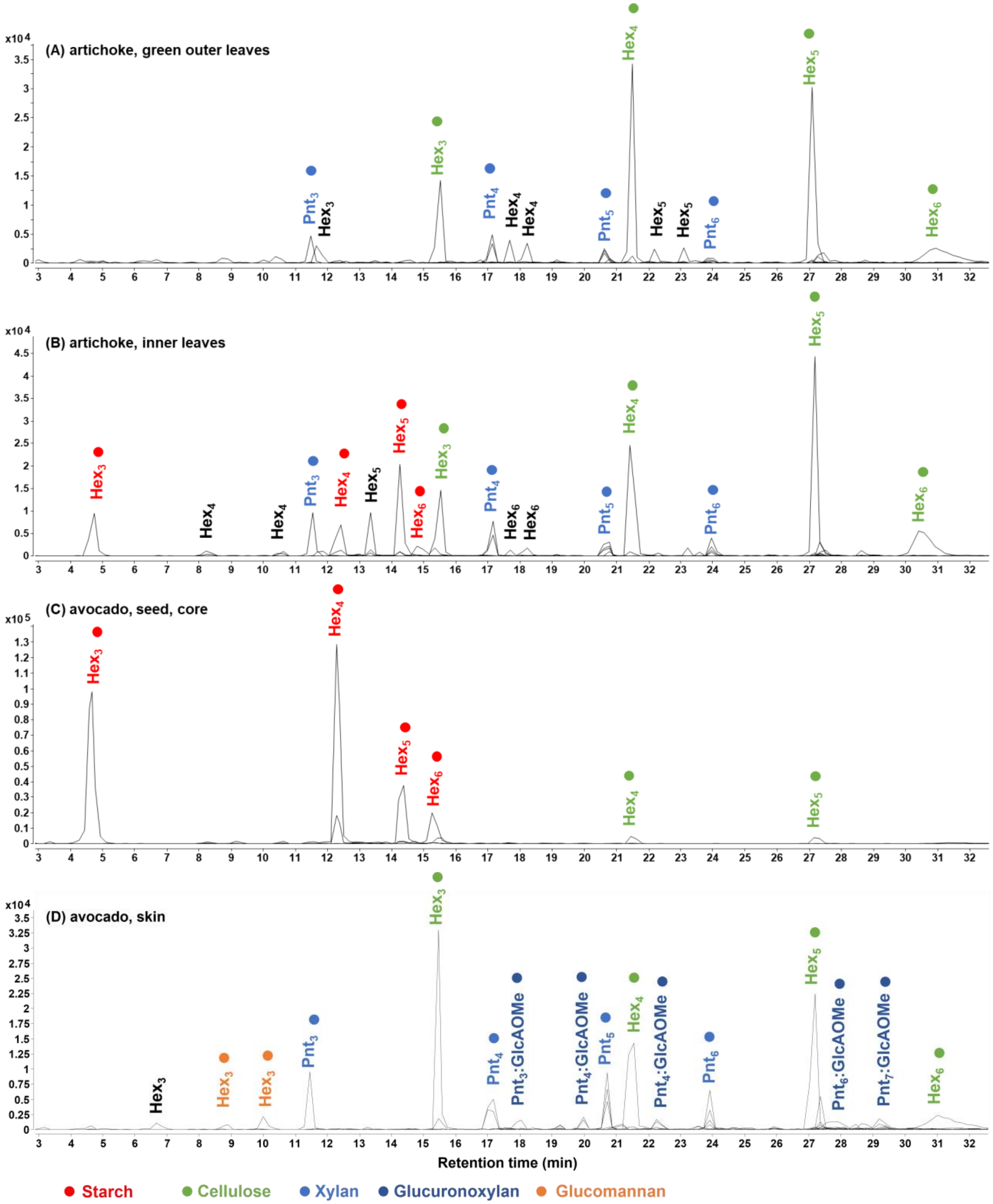

Figure S6. Examples of annotated chromatograms (oligosaccharide profiles) of various food samples. **(A)** green outer leaves from artichoke, **(B)** inner leaves from artichoke, **(C)** avocado seed, and (**D**) avocado skin. Y-axes are in ion counts.
